# Mir-152 Regulates 3T3-L1 Preadipocyte Proliferation and Differentiation

**DOI:** 10.3390/molecules24183379

**Published:** 2019-09-17

**Authors:** Yuan Fan, Mailin Gan, Ya Tan, Lei Chen, Linyuan Shen, Lili Niu, Yihui Liu, Guoqing Tang, Yanzhi Jiang, Xuewei Li, Shunhua Zhang, Lin Bai, Li Zhu

**Affiliations:** 1 College of Animal Science and Technology, Sichuan Agricultural University, Chengdu 611130, Sichuan, China; fanyuan0701@163.com (Y.F.); 18299095425@139.com (M.G.); Tanya_Lee@126.com (Y.T.); chenlei815918@163.com (L.C.); shenlinyuan0815@163.com (L.S.); niulili@sicau.edu.cn (L.N.); tyq003@163.com (G.T.); jiangyz04@163.com (Y.J.); xuewei.li@sicau.edu.cn (X.L.); zhangsh1919@163.com (S.Z.); 2 Farm Animal Genetic Resource Exploration and Innovation Key Laboratory of Sichuan Province, Sichuan Agricultural University, Chengdu 611130, Sichuan, China; 3 Sichuan Province General Station of Animal Husbandry, Chengdu 611130, Sichuan, China; yihuisky@163.com; 4 Institute of Animal Husbandry and Veterinary, Guizhou Academy of Agricultural Science, Guiyang 550005, Guizhou, China

**Keywords:** miR-152, LPL, 3T3-L1 preadipocyte, lipoprotein lipase, obesity

## Abstract

Adipogenesis is a complex biological process and the main cause of obesity. Recently, microRNAs (miRNAs), a class of small endogenous non-coding RNAs, have been proven to play an important role in adipogenesis by the post-transcriptional regulation of target genes. In this current study, we observed an increment of miR-152 expression during the process of 3T3-L1 cell audiogenic differentiation. A functional analysis indicated that the overexpression of miR-152 inhibited pre-adipocyte proliferation and suppressed the expression of some cell cycle-related genes. Moreover, the overexpression of miR-152 promoted lipid accumulation in 3T3-L1 preadipocytes accompanied by increase of the expression of some pro-audiogenic genes. Additionally, a dual-luciferase reporter assay demonstrated lipoprotein lipase (LPL) was a direct target gene of miR-152 during preadipocyte differentiation. Further analysis showed that miR-152 was positively correlated with adipogenesis and intramuscular fat formation in vivo. Taken together, our findings suggest that miR-152 could suppress 3T3-L1 preadipocyte proliferation, whereas it could promote 3T3-L1 preadipocyte differentiation by negatively regulating LPL. The findings indicate that miR-152 might have a therapeutic significance for obesity and obesity-related metabolic syndrome.

## 1. Introduction

Obesity or overweight has emerged as an epidemic which impairs human beings’ health [1]. Moreover, prevalent obesity is associated with type 2 diabetes, hypertension, cardiovascular disease and even certain cancers in both adults and children [2,3]. In the past few decades, researchers have proven that adipose tissue is a subtle endocrine organ and plays an essential role in energy homeostasis. Adipocytes are major bases of adipose tissue, and their proliferation and differentiation are closely related to the function of fat tissue. Simultaneously, in some circumstances, excess caloric intake and the absenting of functional adipocytes leads to ectopic lipid deposition in non-adipose tissue such as the liver, the heart, and muscle [4,5,6]. Additionally, ectopic fat impairs the normal function of organs and further causes insulin resistance [7,8]. Hence, investigating the epigenetic mechanisms of obesity and its consequent ectopic lipid formation may shed light on therapeutic strategies to solve this health issue.

MicroRNAs (miRNAs) are a class of endogenous non-coding RNA which participate in almost every aspect of biological processes by acting as post-transcriptional regulators [9]. Previous reports from other researchers and ourselves have shown that various miRNAs could affect the biology of adipocytes, adipose tissues function, and intramuscular fat formation. For instance, miR-144, miR-10b and miR-143 promote adipocyte differentiation by targeting Krüppel-Like Factor 3 (KLF3) and C-Terminal Binding Protein 2 (CtBP2), Apolipoprotein L6 (Apol6), and Extracellular Signal Regulated Kinase 5 (ERK5), respectively [10,11,12]. Additionally, miR-200b and miR-27b could inhibit adipogenesis by negatively regulating Krüppel-Like Factor 3 (KLF4) and Peroxisome Proliferator-Activated Receptor γ (PPARγ) [13,14]. Furthermore, miR-429 inhibits differentiation and promotes proliferation in porcine intramuscular preadipocytes [15]. miR-152 is usually recognized as a tumor suppressor miRNA in cancers [16]. Studies have shown that miR-152 highly expressed in well-differentiated 3T3-L1 preadipocytes, which suggests that miR-152 may have an effect on adipogenesis [17]. However, in spite of numerous studies, the regulation mechanism of miR-152 in the biology of adipocytes is still unknown.

In this present study, in vitro experiments and in vivo correlative analysis were performed to explore the role of miR-152 in adipogenesis. In the first avenue, we found that miR-152 gradually increased during 3T3-L1 cell differentiation. By transfecting the miR-152 mimic, the miR-152 inhibitor, and the negative control, we demonstrated that miR-152 could inhibit preadipocyte proliferation by modulating E2F transcription factor 3(E2F3), but it could promote 3T3-L1 cell differentiation by targeting lipoprotein lipase LPL. Moreover, a correlative analysis showed that miR-152 was correlated with adipogenesis and intramuscular fat formation in vivo.

## 2. Materials and Methods

### 2.1. Experimental Animals

All the animal care was approved by the Institutional Animal Care and Use Committee of the College of Animal Science and Technology of Sichuan Agricultural University, Sichuan, China, under the permit of No. DKY-B20131403 (Ministry of Science and Technology, China, approved on 15 June 2004). Sixteen Kunming mice (female, 10 weeks) were randomly divided into two groups and treated with a high-fat diet or normal chow (Dossy life science, Chengdu, Sichuan, China) for 8 weeks. All mice had ad libitum access to feed and water and were housed at 22–24 °C under controlled light.

### 2.2. Determination of Intramuscular Fat (IMF)

Briefly, for the determination of intramuscular fat (IMF), the same muscle samples from mice were collected immediately and stored at −20 °C. IMF was determined as the percentage of fat extracted from 2 g of fresh tissue by the traditional Soxhlet petroleum ether extraction method.

### 2.3. Cell Culture and Transfection

3T3-L1 cells and HeLa cells were purchased from the China Infrastructure of Cell Line Resource (Beijing, China) and maintained in DMEM (Gibco, Carlsbad, CA, USA) supplied with 10% FBS (Gibco, Carlsbad, CA, USA) at 5% CO2 and 37 °C. When cell confluence reached 70%–80%, cells were passaged by trypsin with 0.05% EDTA (Gibco, Carlsbad, CA, USA). To induce 3T3-L1 differentiation, cells were planted in 12 wells plates. When the cells reached confluency, the growth medium was changed to a differentiation medium which contained 10% FBS, 1 μM of dexamethasone (DEX, Sigma, Burlington, MA, USA), 0.5 mM of 3-isobutyl-1-methylxanthine (IBMX, Sigma, Burlington, MA, USA), and 10 μg/mL of insulin (Sigma, Burlington, MA, USA). After 3 days of treatment, the medium was switched to the DMEM with 10% FBS and 10 μg/mL of insulin for another two days and subsequently cultured in a culture medium. For transfection, briefly, cells were transfected with 50nM of miR-152 mimic, inhibitor or negative control oligonucleotides supplied by Ribobio (Guangzhou, Guangdong, China) using the lipid carrier Lipofectamine 2000 (Invitrogen, Carlsbad, CA, USA), according to the manufacturer’s instructions. The transfection was carried out every second day, and the medium was changed 8 h after transfection.

### 2.4. RNA Extraction and Quantitative qRT-PCR

Mice sacrificed by cervical dislocation, gonadal fat mass, and gastrocnemius muscle samples were frozen immediately in liquid nitrogen and stored at −80 °C. The total RNA of adipose tissue, muscle tissue, and cell samples were extracted with the RNAiso Plus (TaKaRa, Dalian, Liaoning, China). The PrimeScript™ RT reagent Kit with the gDNA Eraser (TaKaRa Dalian, Liaoning, China) and Mir-X miRNA First-Strand Synthesis Kit (TaKaRa Dalian, Liaoning, China) were used for reverse-transcribing mRNA and miRNA to cDNA, respectively. Quantitative real-time PCR (qRT-PCR) reactions were performed by using a SYBR Premix Ex Taq kit (TaKaRa Dalian, Liaoning, China) and a CFX96 RealTime PCR detection system (Bio-Rad, Hercules, CA, USA). The relative expression levels of mRNA and miRNA were calculated using the 2^−ΔΔct^ method [18]. β-Actin and U6 snRNA were used as internal normalizing controls for mRNA and miRNA, respectively. All PCR primer sequences shown in Supplementary Table S1.

### 2.5. Cell Proliferation Assays

For the Cell Counting Kit 8 (CCK-8) assay, the 3T3-L1 preadipocyte was seeded in 96 plates. The absorbance of cells was detected at the times of 0, 24, 48, and 96 h after the transfection of the miR-152 mimic, the miR-152 inhibitor and the negative control by using Cell Counting Kit 8 (Dojindo, Shanghai, China) according to the manufacturer’s protocol.

For the 5-ethynyl-20-deoxyuridine (EdU) assay, after 36 h of transfection, cells were incubated in 96-well plates supplied with a growth medium containing 50 mM of Edu reagent (RiboBio, Guangzhou, Guangdong, China). After 2 h of EdU incubation, fixation, permeabilization, and staining, sample images were captured using a Nikon TE2000 microscope (Nikon, Tokyo, Japan), and positive cells were counted by Photoshop 6.0.

### 2.6. Oil Red O Staining and Triglyceride Assay

As previously described [11], well-differentiated 3T3-L1 cells were washed with PBS three times, fixed in 4% paraformaldehyde for 1 h, and washed with PBS again. Then, cells were stained with 5% Oil Red O (Sigma, St. Louis, MO, USA) for 1 h, washed twice with PBS, and photographed. For the triglyceride assay, the optical density (OD) values were detected with Multiskan GO (ThermoFisher, Carlsbad, CA, USA) at a wavelength of 510 nm.

### 2.7. Luciferase Reporter Assay

The luciferase reporter plasmids psi-CHECK2™ (Promega, Madison, WI, USA) containing wild-type 3′UTR or mutant-type 3′UTR of LPL were made by TsingKe Biotech (Chengdu, Sichuan, China). The wild-type and mutant LPL 3′UTR were inserted into sites between XhoI and NotI restriction, respectively. HeLa cells were co-transfected with the plasmids combined with the miR-152 mimic or the negative control. After 36 h of transfection, the cells were harvested, and the luciferase activities were measured using the Dual-Glo Luciferase Assay System (Promega, Madison, WI, USA), according to the manufacturer’s instructions.

### 2.8. Statistical Analysis

All quantitative results are presented as mean ± standard deviation (SD). The differences between two groups were analyzed by a Student’s *t*-test. The groups of three were analyzed by a one-way ANOVA. Statistical analyses were conducted using SPSS 20.0 software (IBM, Almond, NY, USA). *p* < 0.05 was considered to be statistically significant.

## 3. Results

### 3.1. miR-152 is Up-Regulated During 3T3-L1 Preadipocyte Differentiation

**Figure 1:**
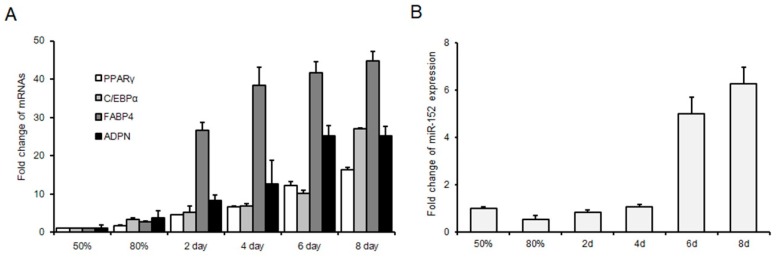
MicroRNA (miR)-152 is up-regulated during 3T3-L1 preadipocyte differentiation. (**A**) The expression of adipogenic markers in 3T3-L1 cells in GM 50% and 80% and DM for two, four, six and eight days. (**B**) The expression of miR-152 in 3T3-L1 cells in GM 50% and 80% and DM for two, four, six and eight days. Results are presented as means ± standard deviation (SD).

To explore the role of miR-152 in adipogenesis, we used 3T3-L1 cells as the cell model. Firstly, we naturally induced 3T3-L1 cell differentiation. The mRNA levels of audiogenic markers were detected. The result showed that these markers were increased gradually during adipogenesis (Figure 1A). Then, the expression level of miR-152 was detected during 3T3-L1 cell proliferation and differentiation by using qRT-PCR. We found that miR-152 is decreased in confluence from 50% to 80% during proliferation and increased gradually from day two to day eight during differentiation (Figure 1B). These results indicated that miR-152 might play an important role in 3T3-L1 proliferation and differentiation.

### 3.2. miR-152 Inhibits 3T3-L1 Preadipocyte Proliferation

**Figure 2:**
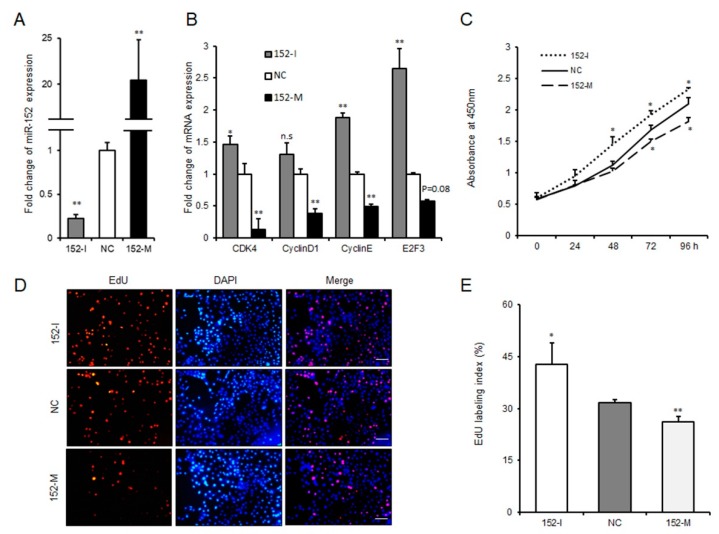
miR-152 inhibits 3T3-L1 preadipocyte proliferation. (**A**) The efficiency of miR-152 overexpression and knockdown. (**B**) The mRNA expression levels of CDK4, cyclin D1, cyclin E, and E2F3 were detected by qRT-PCR after 3T3-L1 cells transfected with the miR-152 mimic, the miR-152 inhibitor or the negative control for 36 h, respectively. (**C**) Cell proliferation were measured by a CCK-8 (Cell Counting Kit-8) assay after 3T3-L1 cells were transfected with the miR-152 mimic, the miR-152 inhibitor or the negative control. (**D**) After transfection with the miR-152 mimic, the miR-152 inhibitor or the negative control for 36 h, cells were fixed for 5-ethynyl-20-deoxyuridine (EdU) staining. (**E**) The proportion of EdU-positive cells are presented. Results are presented as means ± SD. n = 3. * *p* < 0.05; ** *p* < 0.01. Scale = 100 μm.

As a first step to investigate whether miR-152 might regulate the proliferation of 3T3-L1 cells, we transfected cells with the miR-152 mimic, the miR-152 inhibitor or the negative control. As shown in Figure 2A, the expression level of miR-152 was significantly increased or decreased compared to the negative group. Cyclin-dependent kinases (such as CDK4), cyclin D1 and cyclin E have been recognized as key regulators of cell growth and proliferation in mammalian cells [19]. Then, qRT-PCR was performed to evaluate the function of miR-152 on the expression of these genes during 3T3-L1 cell proliferation. Results indicated that the knockdown of miR-152 could remarkably increase CDK4 and cyclin E expression; the overexpression of miR-152 significantly suppressed the expression of CDK4, cyclin D1 and cyclin E (Figure 2B). E2F3 belongs to E2Fs transcription factor family, and previous studies from other researchers and ourselves have shown that E2F3 plays a positive role in adipocyte proliferation and is a target of miR-152 [20,21]. qRT-PCR results also showed that altering miR-152 expression could hinder or promote E2F3 expression (Figure 2B). Additionally, to confirm the function of miR-152 on preadipocyte proliferation, a CCK-8 assay was applied. As shown in Figure 2C, after 48 or 72 h transfection, the growth rate of 3T3-L1 cells was significantly increased or repressed in the miR-152 inhibitor and miR-152 mimic groups compared to the control group, respectively. A 5-ethynyl-20-deoxyuridine (EdU) staining analysis also confirmed this finding. The ratio of EdU-positive cells was significantly increased after the knockdown of miR-152, while it was decreased after the transfection of the miR-152 mimic (Figure 2D,E). Therefore, these results suggest that miR-152 could inhibit 3T3-L1 cell proliferation.

### 3.3. miR-152 Promotes 3T3-L1 Preadipocyte Differentiation

**Figure 3:**
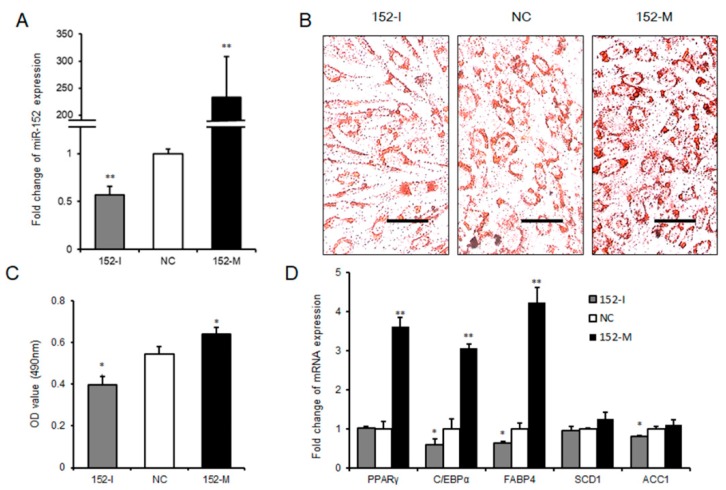
miR-152 promotes 3T3-L1 preadipocyte differentiation. (**A**) The efficiency of miR-152 overexpression and knockdown during 3T3-L1 cell differentiation. (**B**) Intracellular lipid accumulation was measured by Oil Red O Staining on day eight after adipogenic induction. (**C**) The contents of triglycerides in terminally differentiated adipocytes. (**D**) The mRNA expression levels of PPARγ, C/EBPα, FABP4, SCD1, and ACC1 were detected by qRT-PCR after 3T3-L1 cells transfected with the miR-152 mimic, the miR-152 inhibitor or the negative control on day eight; Results are presented as means ± SD. n = 3. * *p* < 0.05; ** *p* < 0.01. Scale = 100 μm.

To further explore the role that miR-152 played in adipogenesis, we transfected 3T3-L1 cells with the miR-152 mimic, the miR-152 inhibitor, or the negative control in succession. The transfection efficiency detected by qRT-PCR is displayed in Figure 3A. Interestingly, as shown in the Oil Red O staining, we found that the knockdown miR-152 significantly impaired the total lipid accumulation in 3T3-L1 cells compared to the negative control. In agreement with this result, the production of lipid drops was increased after overexpressing miR-152 (Figure 3B,C). To further investigate the function of miR-152 in adipogenesis, we detected the expression levels of some adipogenesis regulators and markers related to adipogenesis. Following these findings, the transfection of miR-152 inhibitor significantly decreased adipogenic transcript expression, including CCAAT/Enhancer Binding Protein α (C/EBPα), Fatty Acid-Binding Protein 4 (FABP4), and Acetyl-CoA Carboxylase 1 (ACC1), and the expression of PPARγ, C/EBPa, and FABP4 had higher levels in the miR-152 mimic group when compared to the negative control group (Figure 3D). Taken together, these results indicate that miR-152 could promote 3T3-L1 preadipocyte differentiation.

### 3.4. Lipoprotein Lipase is a Target Gene of miR-152

**Figure 4:**
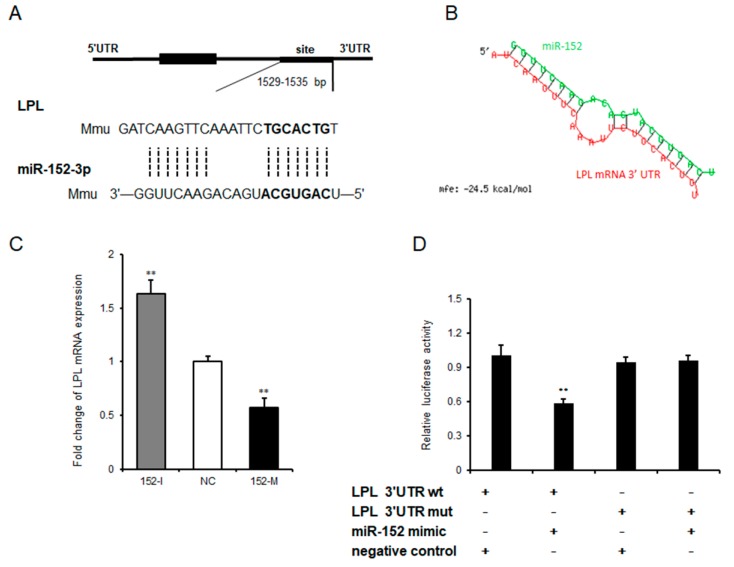
Lipoprotein lipase is a target of miR-152. (**A**) The sequence alignment of miR-152 with 3′-UTR of mouse lipoprotein lipase (LPL) mRNA. The binding site and seed region of miR-152 re indicated in bold. (**B**) The minimum free energy of miR-152 and LPL mRNA was predicted using RNAhybrid. (**C**) The mRNA expression level of LPL was detected by qRT-PCR after 3T3-L1 cells transfected with the miR-152 mimic, the miR-152 inhibitor or the negative control on day eight. (**D**) The repressive effect of miR-152 on the activity of LPL 3′UTR was measured by a dual-luciferase assay. Results are presented as means ± SD. n = 3. * *p* < 0.05; ** *p* < 0.01.

In general, microRNA execute its biological processes through hastening the decay of its target genes by binding to mRNAs’ 3′UTR [22]. To further explore the potential underlying mechanism of how miR-152 regulates adipogenesis, Targetscan was used to predict potential target genes of miR-152. We found that LPL—shown to be important in adipose tissue triglyceride accumulation according to a previous study [23]—is a target of miR-152 Figure 4A. Moreover, the predicted minimum free energy of miR-152 and LPL mRNA 3′UTR was found to be −24.5 kcal/mol by using RNAhybrid [24] (Figure 4B). Next, LPL mRNA levels were detected in 3T3-L1 cells after transfecting the miR-152 mimic, the miR-152 inhibitor or the negative control. qRT-PCR results showed that the transfection of the miR-152 inhibitor significantly increased LPL mRNA expression, and the overexpression of miR-152 showed an opposite effect when compared to the negative control group (Figure 4C). To further validate the inhibitory role of miR-152 on LPL mRNA, we constructed luciferase reporter plasmids containing wild-type (WT) and mutant (MUT) sequences of the LPL 3′UTR region. As seen in Figure 4D, the transfection of the miR-152 mimic significantly suppressed the luciferase activity of plasmids containing the LPL 3′UTR region with miR-152 binding sites, but it did not alter the luciferase activity of plasmids containing the mutant sequence. Collectively, LPL is a bona fide target of miR-152.

### 3.5. miR-152 is Correlated with Adipogenesis and Intramuscular Fat Formation In Vivo

**Figure 5:**
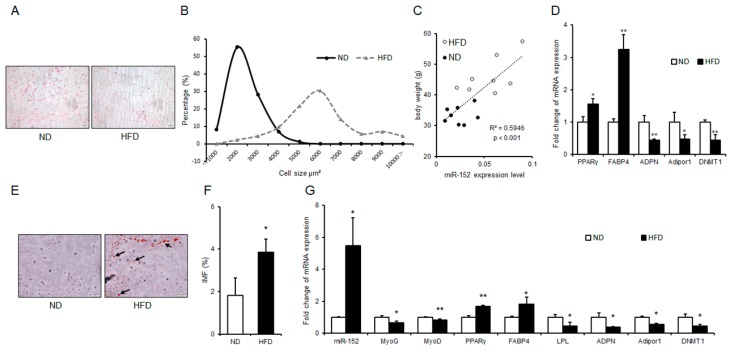
miR-152 is correlated with adipogenesis and intramuscular fat formation in vivo. (**A**) H and E staining for the gonadal fat and sizes of adipose cells were analyzed by using ImageJ. (**B**) The cell size profiling of gonadal fat mass. (**C**) Correlation analysis between miR-152 expression levels in gonadal fat mass and bodyweight of mice, n = 16; (**D**) The mRNA expression levels of PPARγ, FABP4, ADPN, Adipor1 and DNMT1 were detected in the gonadal fat mass of normal diet (ND) mice and high fat diet (HFD) mice; (**E**) Oil Red O staining for intramuscular fat in gastrocnemius muscle of ND mice and HFD mice (arrowheads point to lipid droplets) and (**F**) Intramuscular fat (IMF) levels was detected, n = 3; (**G**) The miRNA expression level of miR-152 and the mRNA expression levels of MyOG, MyOD, PPARγ, FABP4, LPL, ADPN, Adipor1 and DNMT1 were detected in the gastrocnemius muscle of ND mice and HFD mice, n = 3. Results are presented as means ± SD. * *p* < 0.05; ** *p* < 0.01. Scale = 50 μm.

To figure out whether the pro-adipogenic effect of miR-152 is constant in vivo, obese mice were raised and supplied with high-fat diet (HFD). As was expected, H and E staining and cell size profiling showed the sizes of gonadal adipose cells in obese mice were bigger than those in a normal diet (ND) mice (Figure 5A,B). Then, we detected miR-152 levels in the gonadal fat mass of mice. Interestingly, we found that there was a significant positive correlation between the miR-152 levels and body weight of mice (Figure 5C). Previous studies have shown that DNA Methyltransferase 1 (DNMT1) is a target gene of miR-152 and could be a key player in adipogenesis [25,26]. Hence, we performed qRT-PCR to detect mRNA levels in gonadal fat. Results showed the pro-adipogenic transcription of PPARγ and FABP4 was significantly increased in obese mice, while the anti-adipogenic transcription of Adiponectin (ADPN), Adipor1, and DNMT1 was down-regulated (Figure 5D). Previous reports suggested that the excessive intake of caloric and absenting of functional adipocytes causes ectopic lipid deposition in skeletal muscle [5]. As was expected, we found that there was ectopic lipid deposition in obese mice’s gastrocnemius muscle by Oil Red O staining (Figure 5E), and IMF determination further confirmed this result (Figure 5F). Then, we found that miR-152 was highly up-regulated in the gastrocnemius muscle of obese mice compared to ND mice. A subsequent analysis found that the mRNA levels of myogenic gene Myogenin (MyOG) and Myoblast Determination Protein (MyOD), as well as the mRNA levels of anti-adipogenic transcription (ADPN, Adipor1, LPL and DNMT1) were significantly decreased. Expectedly, PPARγ and FABP4 levels were promoted (Figure 5G). These results demonstrated that miR-152 was correlated with adipogenesis in vivo.

## 4. Discussion

**Figure 6:**
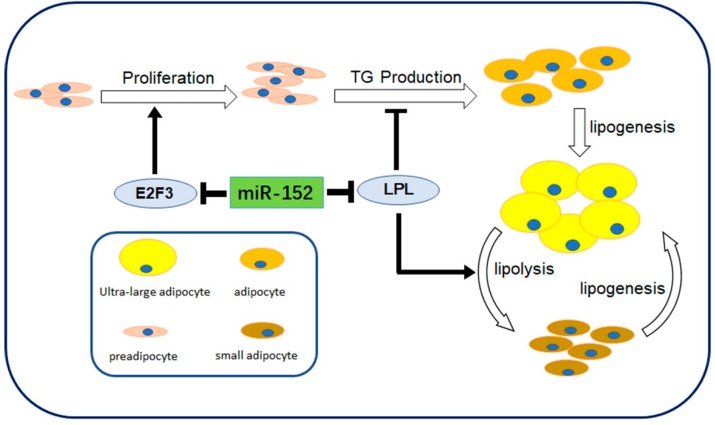
Schematic diagram showing miR-152 roles in 3T3-L1 preadipocyte cells. miR-152 regulates proliferation, triglycerides (TG) production and lipogenesis of small adipocytes by modulating E2F3 and LPL.

miR-152, a member of the miR-148-152 cluster, has been shown to participate in many biological processes. Previous studies have mainly focused on the clinical implications and the tumor suppressor function of miR-152. For instance, miR-152 might be a potential epigenetic biomarker in both gastrointestinal cancers and bladder cancer [27,28]. Moreover, miR-152 could inhibit tumor cell growth in various cancers, such as endometrial cancer and glioblastoma [16,29]. However, the effect of miR-152 in adipogenesis is still unknown. Here, our investigation revealed that miR-152 increased during preadipocyte differentiation. Furthermore, the loss-of-function or the gain-of-function of miR-152 was applied to detect the function of miR-152 in the proliferation and differentiation of preadipocytes, and a dual-luciferase assay was performed to investigate the direct binding relationship between miR-152 and LPL mRNA. Based on our observations, we report that miR-152 could depress the proliferation and promote the differentiation of 3T3-L1 cells by partially modulating E2F3 and LPL, the target genes of miR-152 (Figure 6).

As stated above, the miRNAs are involved in biological processes, usually by canonical seed matches. Lipoprotein lipase (LPL), the downstream target of miR-152 indicated in this study, is a critical hydrolytic enzyme which regulates lipid and lipoprotein metabolism. Lipoprotein lipase plays an essential role in the hydrolysis of core triglycerides (TGs) in chylomicrons and very-low-density lipoproteins (VLDLs) [30,31]. As shown in this study, the transfection of the miR-152 mimic induced an LPL mRNA decrement and subsequently increased lipid storage in cells. Additionally, miR-148a/152 shown to evoke some metabolic syndromes, such as hyperlipidemia, hypercholesteremia, and atherosclerosis [32,33]. Abnormal LPL could induce these metabolic syndromes [34,35]. These findings indicated miR-152 might regulate obesity-associated syndromes by modulating LPL.

The skeletal muscle, liver, and adipose tissue are primary tissues in the whole-body metabolism of mammals. Our previous report showed that miR-152 could impair the function of skeletal muscle cells by inhibiting its proliferation and differentiation [21,36]. Moreover, a study indicated miR-152 up-regulated in fatty liver tissues and cells cultured with Free Fatty Acids (FFAs) and proinflammatory factors [37]. A recent study showed that hepatic Argonaute-2 (Ago2)-deficiency could improve glucose metabolism in conditions of insulin receptor antagonist treatment and high-fat diet challenge, partially by down-regulating miR-152 [38]. Here, we showed miR-152 promotes 3T3-L1 cell adipogenesis and correlates with intramuscular fat formation. Collectively, the evidence from this study suggests that miR-152 affects metabolism not only by regulating liver and skeletal muscle but also by inducing fat tissue adipogenesis. Thus, considering the significant correlation between miR-152 and these metabolic syndromes, miR-152 might serve as a therapeutic target in chronic metabolic disorders, such as diabetes and other obesity-associated sequelae.

## 5. Conclusions

In this current study, we focused on the expression and function of miR-152 both in vitro and in vivo. It was shown that the overexpression miR-152 impaired 3T3-L1 cell proliferation and promoted differentiation, whereas miR-152 knockdown had the opposite effect. We found that LPL is a direct target gene of miR-152. Furthermore, an in vivo correlation analysis indicated miR-152 correlated with adipogenesis and intramuscular fat formation. These results confirmed that miR-152 plays an effect on adipogenesis and obesity. Finally, miR-152 should be considered as a potential therapeutic molecule for obesity and obesity-related metabolism syndrome.

## Supplementary Materials

The supplementary materials are available online, Table S1: Sequences of PCR primers used in this study.
